# OA16 Sjögren’s syndrome with plasma-cell–rich tubulo interstitial nephritis- a challenging autoimmune–haematological overlap

**DOI:** 10.1093/rap/rkag113.016

**Published:** 2026-11-03

**Authors:** Hala Abbas, Mithun Chakravorty

**Affiliations:** Barnsley Hospital, Sheffield, United Kingdom; Barnsley Hospital, Sheffield, United Kingdom

## Abstract

**Introduction:**

A 59-year-old woman with primary Sjögren’s syndrome, plasma-cell dyscrasia, and biopsy-proven tubulointerstitial nephritis presented with progressive fatigue, worsening ankle/knee pain, and prolonged early-morning stiffness. Her renal disease an uncommon extra-glandular manifestation of Sjögren’s had previously responded to corticosteroids but resulted in avascular necrosis, prompting transition to rituximab with subsequent stabilisation of kidney function. Over time she developed intermittent mouth ulcers, hair thinning, and joint tenderness without synovitis. Autoimmune serology showed ANA and anti-Ro positivity, antimitochondrial antibodies, but negative rheumatoid factor, anti-CCP, ANCA, dsDNA, and normal immunoglobulins. Hydroxychloroquine was trialled but poorly tolerated.

**Case description:**

This a 59 years old lady, with past medical history of PBC, no family history of autoimmune disease presented with Sicca symptoms in 2018 - dry mouth, dry eyes, dry flaky skin, no skin rash, joints pain, no neurology symptoms, no Raynaud’s, no mouth/vaginal ulcers, hair loss or cardio/ respiratory symptoms. She developed CKD with no proteinuria, had two biopsies showed significant inflammatory cell infiltrate with a large plasma effect and a paraprotein (diagnosed as tubular interstitial nephritis which seems to be steroids sensitive). Concurrently, she was diagnosed with IgG-kappa plasma-cell dyscrasia and MGUS. Review at the National Amyloidosis Centre excluded systemic amyloidosis or haematological malignancy Autoimmune profile showed ANA positive, anti-Ro 60 positive, and strong positive mitochondrial antibody. Anti-RNP present (doubtful clinically significant as CK was normal). She had received corticosteroids for renal disease but developed avascular necrosis bilaterally and a left hip replacement. Then has started on rituximab at 6 months, then 12 months then 18 months interval, her kidney functions improved and remains stable. Along the course of disease- started to develop tiredness/fatigue, painful ankles with occasional swelling, knees and wrists pain with early morning stiffness in ankles and knees for1-2 hr and settles after movement, occasional mouth ulcers and hair thinning. On examination: pain across knee, wrists and ankles tenderness but no evidence of synovitis. Rheumatoid factor, anti CCP, C-ANCA/P-ANCA and dsDNA, Immunoglobulin and serum/protein electrophoresis all were negative. normal MPO and RP3. Hence has a trial of hydroxychloroquine to help controlling joints symptoms however, this was poorly tolerated due to GI symptoms and headache hence, stopped.

**Discussion:**

This case highlights a rare and challenging overlap of primary Sjögren’s syndrome, plasma-cell dyscrasia, primary biliary cholangitis, and tubulo-interstitial nephritis. Importantly, renal involvement in Sjögren’s syndrome is uncommon, occurring in a minority of patients and most frequently presenting as tubulo-interstitial nephritis. In this case, the plasma-cell–rich infiltrate and paraprotein complicated the diagnostic picture, raising concern for lymphoproliferative disease and necessitating haematology and amyloidosis review. The absence of systemic amyloidosis and stable MGUS helped narrow the differential to autoimmune-mediated interstitial nephritis. Renal involvement in Sjögren’s is uncommon but well-documented, typically presenting as interstitial nephritis with minimal proteinuria. Her biopsy findings, steroid responsiveness, and subsequent rituximab-mediated stabilisation align with this phenotype. The coexistence of PBC and Sjögren’s is also well recognised, often accompanied by fatigue, complex autoantibody profiles, and increased risk of MGUS or lymphoproliferative evolution. Musculoskeletal symptoms evolved gradually, with inflammatory-type stiffness but no synovitis and negative rheumatoid markers. This pattern is consistent with Sjögren’s-related arthralgia rather than rheumatoid arthritis. Hydroxychloroquine intolerance limited first-line management, highlighting therapeutic challenges in multisystem disease. Rituximab played a central role, improving renal function and providing partial control of systemic symptoms. Its use aligns with evidence supporting B-cell depletion in severe extra glandular Sjögren’s manifestations, including renal involvement. The development of steroid-induced avascular necrosis underscores the importance of steroid-sparing strategies.

**Key learning points:**

• Renal involvement in Sjögren’s is uncommon but can present as plasma-cell–rich tubulo-interstitial nephritis.

• Rituximab is an effective steroid-sparing agent for severe extraglandular disease.

• Coexisting PBC and Sjögren’s requires careful monitoring.

• Persistent arthralgia with negative rheumatoid markers may reflect Sjögren’s-related inflammatory pain rather than rheumatoid arthritis

• Multidisciplinary collaboration is essential in complex autoimmune–haematological overlap syndromes.

